# Influence of Molecular Design on the Targeting Properties of ABD-Fused Mono- and Bi-Valent Anti-HER3 Affibody Therapeutic Constructs

**DOI:** 10.3390/cells7100164

**Published:** 2018-10-11

**Authors:** Mohamed Altai, Charles Dahlsson Leitao, Sara S. Rinne, Anzhelika Vorobyeva, Christina Atterby, Stefan Ståhl, Vladimir Tolmachev, John Löfblom, Anna Orlova

**Affiliations:** 1 Department of Immunology, Genetics and Pathology, Uppsala University, 751 85 Uppsala, Sweden; anzhelika.vorobyeva@igp.uu.se (A.V.); christina.atterby@igp.uu.se (C.A.); Vladimir.tolmachev@igp.uu.se (V.T.); 2 Department of Medicinal Chemistry, Uppsala University, 751 23 Uppsala, Sweden; sara.rinne@ilk.uu.se (S.S.R.); anna.orlova@ilk.uu.se (A.O.); 3 Department of Protein Science, School of Engineering Sciences in Chemistry, Biotechnology and Health, KTH Royal Institute of Technology, 106 91 Stockholm, Sweden; chdl@kth.se (C.D.L.); stefans@biotech.kth.se (S.S.); lofblom@kth.se (J.L.); 4 Science for Life Laboratory, Uppsala University, 752 37 Uppsala, Sweden

**Keywords:** HER3, affibody, molecular design, therapy

## Abstract

Overexpression of human epidermal growth factor receptor type 3 (HER3) is associated with tumour cell resistance to HER-targeted therapies. Monoclonal antibodies (mAbs) targeting HER3 are currently being investigated for treatment of various types of cancers. Cumulative evidence suggests that affibody molecules may be appropriate alternatives to mAbs. We previously reported a fusion construct (3A3) containing two HER3-targeting affibody molecules flanking an engineered albumin-binding domain (ABD_035_) included for the extension of half-life in circulation. The 3A3 fusion protein (19.7 kDa) was shown to delay tumour growth in mice bearing HER3-expressing xenografts and was equipotent to the mAb seribantumab. Here, we have designed and explored a series of novel formats of anti-HER3 affibody molecules fused to the ABD in different orientations. All constructs inhibited heregulin-induced phosphorylation in HER3-expressing BxPC-3 and DU-145 cell lines. Biodistribution studies demonstrated extended the half-life of all ABD-fused constructs, although at different levels. The capacity of our ABD-fused proteins to accumulate in HER3-expressing tumours was demonstrated in nude mice bearing BxPC-3 xenografts. Formats where the ABD was located on the C-terminus of affibody binding domains (3A, 33A, and 3A3) provided the best tumour targeting properties in vivo. Further development of these promising candidates for treatment of HER3-overexpressing tumours is therefore justified.

## 1. Introduction

Extensive research over the last decades has led to a better understanding of tumour biology and specific mechanisms of cancer development [1]. It has been demonstrated that cancer hallmarks such as resistance to apoptosis, invasiveness, and high proliferative rate are regulated and controlled by a sophisticated intracellular system of signalling cascades. These signalling pathways are often triggered by aberrantly expressed surface receptors such as receptor tyrosine kinases (RTKs). RTKs are transmembrane receptors sub-classified into 17 different classes that all have an intracellular domain that activates cellular downstream signalling pathways after interaction of the extracellular domain with an appropriate ligand [2]. Growth factors, cytokines, and hormones serve as natural ligands for receptor activation. Dysregulation of any element in the ligand–receptor–signalling chain can be a driving force in sustaining signalling. Advancements in our understanding of the mechanisms underlying cancer hallmarks have led to the design and development of a new class of anti-cancer drugs that are more cancer-specific. They are based on targeting of particular elements in the ligand–receptor–signalling chain. Monoclonal antibodies (mAbs) constitute the most well-studied class of targeting drugs. Today over 50 antibodies are approved by the US Food and Drug Administration (FDA), and many more are under development [3]. Many of these approved mAbs are directed against cancer. The mechanisms of action of mAbs as anti-cancer agents include: preventing ligand–receptor interactions (e.g., bevacizumab, which blocks vascular endothelial growth factor (VEGF) from binding to receptors), downregulating receptor expression (e.g., trastuzumab for treatment of human epidermal growth factor receptor type 2 (HER2)-positive tumours), preventing receptor dimerization (e.g., pertuzumab), and recruitment of the endogenous immune system (for example through activation of antibody-dependent cell-mediated cytotoxicity (ADCC)). In addition, mAbs can be utilized as vehicles to deliver toxic payloads to targeted cells [4].

Human epidermal growth factor receptor type 3 (HER3), an RTK belonging to the HER family, is a key player in many types of cancers. Enhanced expression of these receptors correlates with poor prognosis and disease progression [5,6]. Today, there are no approved protein-based drugs specifically targeting HER3. Currently, two mAbs (patrimumab and seribantumab) are being evaluated in late-phase clinical trials for treatment of several HER3-expressing cancers (National Clinical Trials: NCT02134015, NCT02387216). However, mAbs are not in all cases ideal for therapeutic applications. For example, the large size of mAbs hampers efficient extravasation and reduces tissue penetration, and stability issues sometimes occur [7]. Moreover, mAb production is challenging and expensive (especially in terms of good manufacturing practice compliance).

One possible alternative to mAbs is the use of engineered scaffold proteins (ESPs) [8]. ESPs are sometimes referred to as antibody-mimetics where the scaffold protein is composed of a constant region (which stabilizes the overall protein folding) and variable regions that mediate its binding to a specific target. Several features of non-immunoglobulin-based scaffolds might be attractive for drug applications: robust structure, defined chemical composition, small size, multimerization possibilities, and relatively low production costs [9,10,11]. Moreover, available clinical reports have clearly demonstrated the safety, low immunogenicity, and efficacy of ESP-based therapeutics [12]. Many ESPs permit high production yield in bacterial expression systems. Over 50 different scaffolds have been reported within the last two decades; however, only a limited number of them have undergone thorough development [12].

Affibody molecules are developed by combinatorial protein engineering of the 58-residue (molecular weight (MW) ≈ 7 kDa) protein A-derived Z domain [13,14]. This class of targeting agents has been successfully evaluated in the clinic for molecular recognition in both diagnostic and therapeutic applications. Affibody-based HER2 binders have been used in the clinic for visualization of HER2 expression in metastatic breast cancer [15,16], C5 complement binders have been used for treatment of inflammatory diseases (NCT02083666), and interleukin-17A (IL-17A) binders have been used for treatment of psoriasis (NCT02690142). So far, the accumulated evidence from these clinical trials suggests affibody molecules to be efficacious and safe in humans.

We have reported the selection of an anti-HER3 affibody molecule with affinities to HER3 in the picomolar range [17]. It was demonstrated that anti-HER3 affibody molecules blocked binding of the natural ligand heregulin and the anti-HER3 antibody seribantumab, suggesting that the affibody recognized an overlapping epitope on the receptor [18,19,20,21]. Moreover, it also inhibited heregulin-induced cancer cell growth in vitro by suppressing phosphorylation [18]. A common observation from biodistribution studies of radiolabelled anti-HER3 affibody molecules was the rapid clearance from the blood via kidneys [22]. This is typical for affibody molecules as they have a size below the kidney cut-off (<60 kDa) [11]. One strategy to extend in vivo half-life is based on genetic fusion of affibody molecules to an engineered albumin-binding domain (ABD) [23]. ABD_035_ (46 amino acids, MW ≈ 5.2 kDa) binds to human serum albumin with femtomolar affinity [24]. ABD-mediated half-life extension has been explored for affibody molecules, antibody fragments, and peptides, and has efficiently increased the plasma half-life of fused conjugates up to around the half-life that is reported for endogenous albumin (i.e., around 30 h in mice and 12–17 days in humans) with no immunogenicity issues [11,23,25].

It has been proposed that multivalent constructs could provide increased therapeutic potential compared to monomeric analogues. Indeed, in vitro the bi- and trivalent HER3-specific affibody molecules were significantly more efficient in blocking phosphorylation of HER3 and inhibiting cellular activity and proliferation compared to the monovalent control [20,26]. We demonstrated that a bivalent construct based on an anti-HER3 affibody molecule fused with ABD, 3A3, also efficiently inhibited growth of HER3-expressing cells in vitro, had prolonged in vivo half-life, delayed growth of HER3-expressing BxPC-3 xenografts in mice, and was equipotent to seribantumab (MM-121) [27,28]. Importantly there was no observable toxicity after multiple administrations of the construct [28].

**Figure 1:**
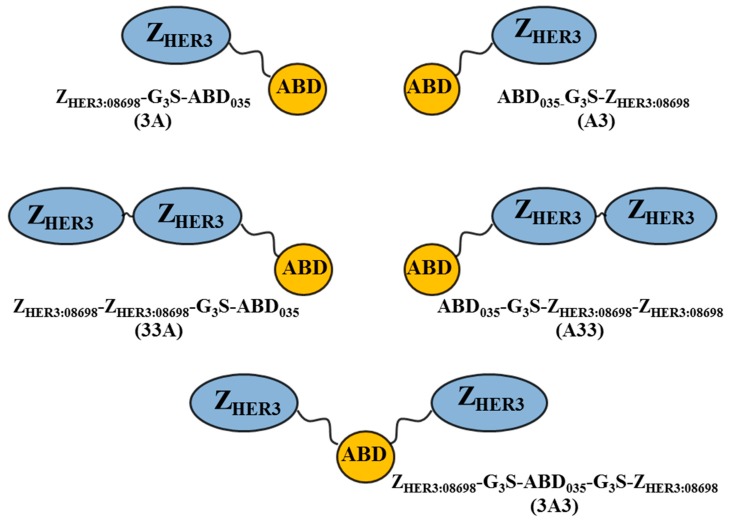
Schematic representation of anti-HER3 affibody molecules flanking ABD with different orientations. ABD: albumin-binding domain; HER3: human epidermal growth factor receptor type 3.

Earlier studies have demonstrated a strong influence of molecular design (sequence of binding domains, valency, composition of linkers and termini) on in vivo targeting and biodistribution properties of targeting agents [29,30,31,32]. Dependent on design, constructs might interact differently with targeted as well as non-targeted tissues. Careful optimization of the molecular design may therefore help to find drugs with desired properties. Such effects become additionally important when the drug efficacy depends on binding to two different targets synchronously. We therefore hypothesized that the relative position of the building molecular blocks of the ABD-fused HER3-targeting affibody molecules (i.e., HER3-binding affibody and albumin-binding moiety) may have a profound influence on targeting properties. In the present study, we have designed and explored a series of novel formats of anti-HER3 affibody molecules flanking ABD using similar molecular blocks: anti-HER3 affibody molecule Z_HER3:08698_, albumin-targeting ABD_035_, and a G_3_S linker. We included five constructs with one or two HER3-targeting arms fused to ABD with different orientations (Figure 1): two monovalent constructs, Z_HER3:08698_-G_3_S-ABD_035_ and ABD_035_-G_3_S-Z_HER3:08698_ (designated as 3A and A3, respectively), and three bivalent constructs, Z_HER3:08698_-Z_HER3:08698_-G_3_S-ABD_035_, Z_HER3:08698_-G_3_S-ABD_035_-G_3_S-Z_HER3:08698_, and ABD_035_-G_3_S-Z_HER3:08698_-Z_HER3:08698_ (designated as 33A, 3A3, and A33, respectively). For investigation of in vitro and in vivo targeting and biodistribution, all constructs were site-specifically conjugated with a 1,4,7,10-tetraazacyclododecane-1,4,7,10-tetraacetic acid (DOTA) chelator and radiolabelled with the residualizing radionuclide ^111^In. For simplicity, the DOTA-conjugated constructs are further designated as 3A, A3, 33A, 3A3, and A33.

It is important to mention that the bivalent construct 3A3 used in the current study comprises similar molecular blocks to those reported by Bass et al. [27]. However, the newly designed construct used in this study differs in that a shorter G_3_S linker is used between the binding moieties. Shorter linker lengths have the potential to result in higher production yields and lower susceptibility to serum proteases. This makes 3A3 a very similar but slightly smaller bivalent construct compared to the previously reported variant. 

Our results reveal that the relative position of the building molecular blocks of the ABD-fused HER3-targeting affibody molecules has an impact on internalization rate, half-life in blood, and tumour targeting.

## 2. Materials and Methods

### 2.1. Production of Constructs, Chelator Conjugation, and Product Purification

Genes for the five constructs were synthesized and subcloned into a pET26b(+) vector (Thermo Scientific, Chicago, IL, USA). The plasmids were transformed into BL21*(DE3) Escherichia coli (*E. coli)* (Thermo Fisher Scientific, Chicago, IL, USA) using a standard heat-shock protocol. Protein production proceeded overnight at 25 °C after induced expression with 100 μM isopropyl β-d-1-thiogalactopyranoside (IPTG) at an optical density measured at a wavelength of 600 nm (OD_600_) of 0.8. Following cell lysis with French press, the proteins were recovered with affinity chromatography using human serum albumin (HSA) immobilized to Sepharose matrix as a ligand. TST buffer (25 mM Tris-HCl, 1 mM EDTA, 200 mM NaCl, 0.05% Tween, pH 8.0) was used as running buffer, with ammonium acetate (5 mM, pH 5.5) for washing followed by elution with acetic acid (0.5 M, pH 2.8) and subsequent freeze-drying. 

The freeze-dried proteins were dissolved in ammonium acetate (20 mM, pH 5.5) and reduced with a molar concentration of TCEP (tris(2-carboxyethyl)phosphine) equal to the protein concentration for 30 min at 37 °C. The proteins were incubated at 37 °C for 90 min with a 10-fold molar excess of maleimide–DOTA (CheMatech, Dijon, France) for site-specific conjugation to a C-terminal cysteine on the constructs. Reverse-phase high performance liquid chromatography (RP-HPLC) (Agilent Technologies, Santa Clara, CA, USA) was used for purification following DOTA-conjugation as described previously [27].

### 2.2. Characterization of the Conjugated Proteins

The purity of the constructs was determined using RP-HPLC and an analytical Zorbax 300SB-C18 column (Agilent Technologies, Santa Clara, CA, USA) with a 25–45% acetonitrile elution gradient over 20 min with a flow rate of 1 mL/min.

Circular dichroism spectroscopy was performed using a Chirascan spectropolarimeter (Applied Photophysics, Surrey, UK) with an optical path length of 1 mm in order to analyse the alpha-helical content, thermal stability, and refolding capacity of the constructs at a concentration of 0.25 mg/mL. The thermal stability was evaluated by measuring the change in ellipticity at 221 nm during heating (5 °C/min) from 20 to 90 °C. The melting temperatures (T_m_) were approximated from the data acquired from variable temperature measurements (VTM) by curve fitting using a Boltzmann Sigmoidal model (GraphPad Prism, version 7, GraphPad Software, La Jolla, CA, USA). The refolding capacity was assessed by comparing spectra obtained from measurements at wavelengths in the range of 195–260 nm at 20 °C, before and after thermal denaturation.

Electrospray ionization mass spectrometry (ESI-MS) with a 6520 Accurate-Mass Q-TOF LC/MS apparatus (Agilent Technologies) was used for confirmation of the molecular masses of the purified constructs.

### 2.3. Affinity Determination

The concomitant binding of the constructs to human HER3 (Sino Biological, Wayne, PA, USA) was investigated with a capture setup on a BIAcore T200 system (GE Healthcare, Princeton, NJ, USA) using a CM5 sensor chip with three immobilization levels of HSA (two surfaces with 550 response units (RU) and one with 2000 RU). The constructs were captured on the surfaces whereupon HER3 was injected in a multi-cycle setup using five concentrations of HER3 (3.125, 6.25, 12.5, 25, and 50 nM). The acquired sensorgrams were analysed using a Langmuir 1:1 kinetic model. In addition, the binding affinity to HSA was investigated, using the same sensor chip and multi-cycle setup. Four concentrations of the constructs (1.5625, 3.125, 6.25, and 12.5 nM) were injected in duplicates and allowed to dissociate from the surface. The sensorgrams acquired from the surface immobilized with 2000 RU were analysed using a Langmuir 1:1 kinetic model.

### 2.4. Radiolabelling of Constructs with Indium-111 and Stability Test of Radiolabelled Constructs

^111^In-indium chloride was purchased from Covidien (Petten, The Netherlands). High-quality Milli-Q water (resistance higher than 18 MΩ/cm) was used for preparing solutions. To work in metal free conditions the buffers were purified and incubated with Chelex 100 resin (Bio-Rad Laboratories, Hercules, CA, USA) overnight. Radiolabelling of the constructs with ^111^In was completed according to a previously reported protocol adapted from Bass et al. [27]. Labelling yields were measured using silica-impregnated glass fibre sheets for instant Thin Layer Chromatography (iTLC) with a 0.2 M citric acid mobile phase. Conjugates were purified from any non-bound ^111^InCl_3_ by using size exclusion NAP-5 columns (GE-Healthcare, Uppsala, Sweden) preequilibrated with phosphate buffered saline (PBS).

To evaluate the labelling stability of the labelled constructs, an EDTA-challenge test was performed. Two samples of each radiolabelled construct were diluted with a 500-fold molar excess of the disodium salt of EDTA in water. The samples were kept at room temperature and after 1 h incubation all samples were analysed using radio-iTLC.

### 2.5. In Vitro Studies

The HER3-expressing human cell lines BxPC-3 (primary pancreatic adenocarcinoma), DU-145 (human prostate cancer), MCF-7 (breast cancer cell line), and LS-174T (colon cancer) were used (American Type Culture Collection, ATCC via LGC Promochem, Borås, Sweden). The cells were cultured in Roswell Park Memorial Institute (RPMI) medium (Flow, Irvine, UK) supplemented with 10% foetal bovine serum (Sigma-Aldrich, St. Louis, MO., USA), 2 mM l-glutamine, and a mixture of penicillin 100 IU/mL and 100 µg/mL streptomycin (PEST, Biokrom Kg, Berlin, Germany). HER3 expression for MCF-7 and DU-145 cells was measured using the same method as for BxPC-3 and LS-174T [21].

### 2.6. Inhibition of Phosphorylation

A human Phospho-HER3 ELISA assay was performed on BxPC-3 and DU145 cells according to a previously published protocol [28]. To prepare samples for the ELISA assay, cells were treated with the affibody constructs (200 nM, 10 min at 37 °C). Starvation media was used to equalize volume in positive and negative controls. Thereafter, 4 nM of heregulin was added to the dishes containing the affibody construct and to the positive control; starvation media was added to the negative control. After incubation for 10 min at 37 °C cells were placed on ice, media was removed, and cells were washed twice in ice-cold PBS. Cells were incubated for 5 min on ice with 400 µL of lysis buffer (20 mM Tris-HCl, 150 mM NaCl, 1 mM Na_2_EDTA, 1 mM EGTA, 1% Triton 2.5 mM sodium pyrophosphate, 1 mM β-glycerophosphate, 1 mM Na_3_VO_4_, and 1 µg/mL leuceptin (Cell Signaling Technology, Danvers, MA, USA)), additionally supplied with 1 mM activated sodium orthovanadate (BioVision, City, CA, USA) and phosphatase inhibitor cocktail (Roche, Mannheim, Germany). The lysed cells were scraped from the dish, collected, and sonicated for 45 s, before being centrifuged for 10 min (14,000 rpm, 4 °C). The supernatant was collected and used in the Phospho-HER3 ELISA.

A Human Phospho-ErbB3/HER3 ELISA kit (DuoSet® IC) was purchased from R&D Systems (R&D Systems, Inc., Minneapolis, MN, USA) and used according to manufacturer’s instructions. Briefly, 96-well plates were coated with capture antibody (4 µg/mL in PBS) and incubated overnight. All incubations were done at room temperature. Wells were washed with 0.05% Tween^®^ 20 (Sigma-Aldrich, St. Louis, MOCity, USA) in PBS and blocked with 1% bovine serum albumin (BSA) in PBS for 2 h. Thereafter, 100 µL of respective cell lysate was added to each well in quadruplicates. The plate was incubated for 2 h, aspirated, washed and anti-phospho-tyrosine-HRP diluted in 0.05% Tween® 20, 1% BSA in PBS after manufacturer’s instruction was added. After 2 h the wells were washed and 100 µL substrate solution (1:1 mixture of H_2_O_2_ and tetramethylbenzidine, R&D systems) was added to the wells. Following 15 min of incubation (protected from direct light) the reaction was stopped by adding 50 µL H_2_S_4_ (1 M). Absorption in each well was measured at 450 nm.

### 2.7. In Vitro Specificity

The specificity of the HER3 affibody ABD-fused conjugates was tested on BxPC-3 and DU-145 cells in vitro. A 100 pM solution of each radiolabelled conjugate was added to a set of cell dishes (ca. 1 × 10^6^ cells/dish, 35 mm, *n* = 3). For blocking, an excess of the non-radiolabelled anti-HER3 affibody molecule, Z_HER3:08698_, was added to an additional set of petri dishes 10 min prior to the addition of the respective conjugate. Cells were then treated as described in detail previously [27,28].

### 2.8. Cellular Processing and Internalization

To compare the internalization rate of labelled constructs by different cell lines (BxPC-3, DU145, LS174T, and MCF-7), a continuous incubation method, developed earlier, was used [33]. Briefly, 1 × 10^6^ cells/dish (*n* = 3) were incubated with 100 pM of the respective radiolabelled compounds at 37 °C, in 5% CO_2_. At predetermined time points after the start of incubation, cells in three dishes were analysed for membrane-bound as well as internalized radioactivity. All activity fractions were measured in an automated γ-spectrometer.

### 2.9. In Vivo Studies 

All animal experiments were planned and performed in accordance with national legislation on laboratory animals’ protection and were approved by the Ethics Committee for Animal Research in Uppsala, Sweden (animal permission C143/14, approved 16 September 2014).

Comparative biodistribution studies were performed in female Balb/c nu/nu mice. Mice were implanted with BxPC-3 (5 × 10^6^ cells) in the left hind leg three weeks prior to the start of the experiment. The average animal weight was 19 ± 1 g, and the average tumour weight was 0.3 ± 0.1 g at the start of the experiment. Mice were sorted into five groups (*n* = 12). Each group of animals was injected intravenously with the corresponding radiolabelled anti-HER3 construct. The injected activity was 20 kBq/mouse (100 μL 2% BSA in PBS containing 100-fold molar excess EDTA). The injected protein dose was 2 nmole: 40 µg/mouse for bivalent 33A, 3A3, and A33, and 26 µg/mouse for monovalent 3A and A3. At predetermined time points (1, 4 and 24 hpost injection.) mice were anaesthetized with an excess dose of Rompun/Ketalar mixture (Ketalar (ketamine): 10 mg/mL, Pfizer AB, Stockholm, Sweden; Rompun (xylazine): 1 mg/mL, Bayer AG, Leverkusen, Germany) and euthanized through cervical dislocation. Organs were collected, weighed and their activity content was measured using an automated γ-counter. Organ activity uptake was represented as percentage of injected dose per gram (%ID/g). 

## 3. Results

### 3.1. Production of Constructs, Chelator Conjugation and Product Purification

The mono- and bi-valent ABD-fused HER3-binding constructs were purified using HSA affinity chromatography, coupled to maleimide-DOTA and subjected to RP-HPLC for purification. The purity of the constructs, as determined by RP-HPLC, exceeded 96% for all five proteins (Figure S1).

### 3.2. Characterization of the Conjugated Proteins

Molecular mass determination with ESI-MS confirmed a concordance with the theoretical masses 12670.9 and 19601.6 Da for DOTA-conjugated monovalent and bivalent constructs, respectively (Table 1 and Figure S2). However, the constructs with N-terminally fused ABD (A3 and A33) exhibited a mass deviation of 131.2 Da, typically corresponding to non-processed N-terminal methionine. Furthermore, a smaller additional peak was observed, particularly for 3A3 and A33, which is likely due to a certain degree of unspecific coupling of the DOTA chelator.

The alpha-helical content, thermal stability and refolding of the DOTA-conjugated constructs were investigated with circular dichroism spectroscopy. The thermal denaturation curves for the constructs are shown in Figure S3A and the associated melting temperatures are presented in Table 1. The respective transitions in unfolding for the individual domains could not be resolved, indicating that the transitions are overlapping and the calculated melting temperatures thus represent an approximate average for the entire constructs. Following thermal denaturation, complete refolding of the constructs was evident from comparison of spectra obtained at 20 °C before and after denaturation, with the exception of a small shift in helicity for 3A3 and A33 (Figure S3B).

### 3.3. Affinity Determination

**Figure 2:**
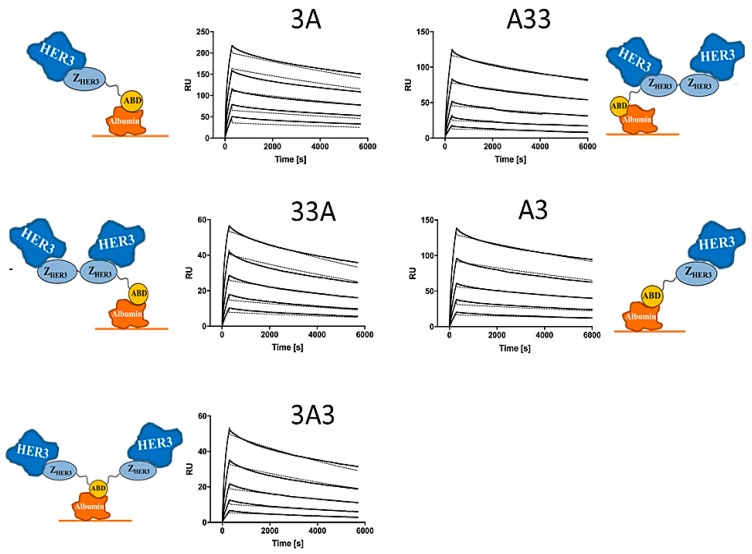
Representative experimental sensorgrams (solid) with fitted curves (dashed) from surface plasmon resonance (SPR) analysis for different ABD-fused anti-HER3 affibody constructs conjugated with a 1,4,7,10-tetraazacyclododecane-1,4,7,10-tetraacetic acid (DOTA) chelator at the C-terminus. Constructs were captured on immobilized HSA and subsequently subjected to five concentrations of human HER3 (3.125, 6.25, 12.5, 25, and 50 nM). Monovalent affinities to HER3, based on a Langmuir 1:1 model, are presented in Table 1.

Kinetic data acquired from surface plasmon resonance (SPR) analysis is presented in Table 1 as the average of triplicates. All DOTA-conjugated constructs demonstrated high affinity to HER3 receptors in the low nanomolar range. K_D_ values refer to the monovalent affinity for human HER3 according to a Langmuir 1:1 model. Representative sensorgrams with fitted curves for each construct are shown in Figure 2. Please note that the interactions were measured in a capture assay, comprising immobilized albumin on the surface followed by consecutive injections of the constructs and HER3. The determined affinities for HER3 are thus for the constructs simultaneously bound to albumin, which more closely represent the situation in vivo. To verify that the affinity for albumin was not dramatically affected by fusion to the affibody molecules, we performed an SPR analysis of the interaction of all constructs, respectively, to albumin. All constructs demonstrated subnanomolar affinity for albumin. The data is presented in Table 1, and the sensorgrams are shown in Figure S4.

### 3.4. Radiolabelling of Constructs with Indium-111 and Stability Test

All DOTA-conjugated anti-HER3 constructs were successfully labelled with ^111^In, though with different labelling yields (Table 2). The radiochemical purity after size exclusion chromatography was over 95% for all conjugates. Challenge with a 500-fold molar excess of EDTA did not show any measurable release of ^111^In when analysed using iTLC, as >95% of the activity was associated to the protein (Table 2).

### 3.5. Inhibition of Phosphorylation

**Figure 3:**
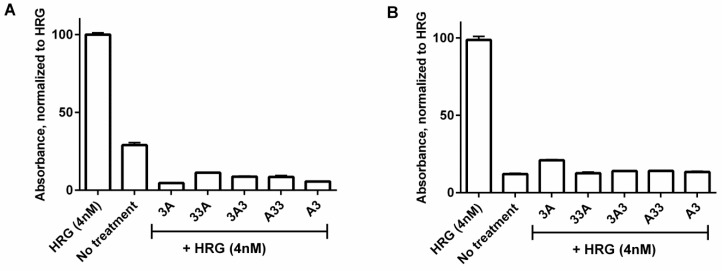
Inhibition efficacy. The relative levels of phosphorylated HER3 for BxPC-3 (**A**) and DU-145 (**B**) cells treated with 4 nM heregulin. Signal was measured using Phospho-HER3 ELISA assay and normalized to the signal of the positive control. Cells were treated either with 4 nM of heregulin or 200 nM of affibody construct, followed by stimulation with 4 nM heregulin. For negative control, cells were left untreated. Data are presented as an average ± SD (*n* = 3).

Human phospho-HER3 ELISA was performed to investigate the influence of the affibody constructs on ligand-induced phosphorylation of HER3 in BxPC-3 and DU-145 cells (Figure 3). In both cell lines, stimulation of HER3 with the natural ligand heregulin showed a significant increase in signalling compared to the negative control, reflecting increased levels of phosphorylated HER3. Cell treatment with the affibody constructs prior to stimulation with heregulin resulted in a significantly lower level of phosphorylated HER3 than in the positive, heregulin-treated control. For BxPC-3 cells, the signalling from the groups treated with affibody constructs was below the level of the negative control. For DU-145 cells with lower HER3 expression level, the signal of the affibody-treated groups matched the signalling of the unstimulated negative control.

### 3.6. In Vitro Specificity

**Figure 4:**
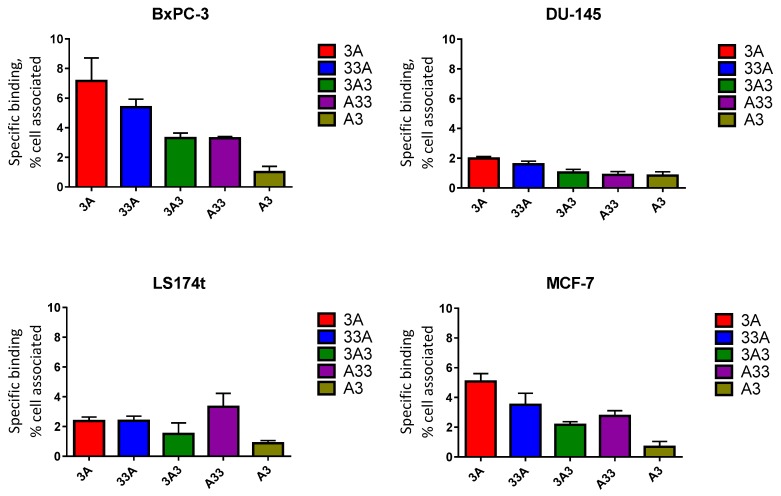
Specific binding (total binding subtracted with the binding after receptor saturation) of different ^111^In-labelled ABD-fused anti-HER3 affibody molecules to HER3-expressing cells (BxPC-3, DU-145, LS174T, and MCF-7) in vitro. Data are presented as an average ± SD (*n* = 3) for the percentage of cell-bound radioactivity from totally added radioactivity.

The binding of all radiolabelled constructs to HER3-expressing cells was significantly reduced (*p* < 0.05) when cells were preincubated with an excess-fold of the non-labelled anti-HER3 affibody molecule Z_HER3:08698_. The specific binding (total binding subtracted with the binding after receptor saturation) of different constructs to HER3-expressing cells is presented in Figure 4.

### 3.7. Cellular Processing and Internalization

The estimated HER3 expression was (15 ± 2) × 10^3^ receptors/cell for MCF-7 cells (high) and (2.8 ± 3) × 10^3^ receptors/cell for DU-145 cells (low). Reported HER3 expression was (12 ± 2) × 10^3^ receptors/cell for BxPC-3 cells (high) and (8.0 ± 0.6) × 10^3^ receptors/cell for LS174T cells (moderate) [21].

**Figure 5:**
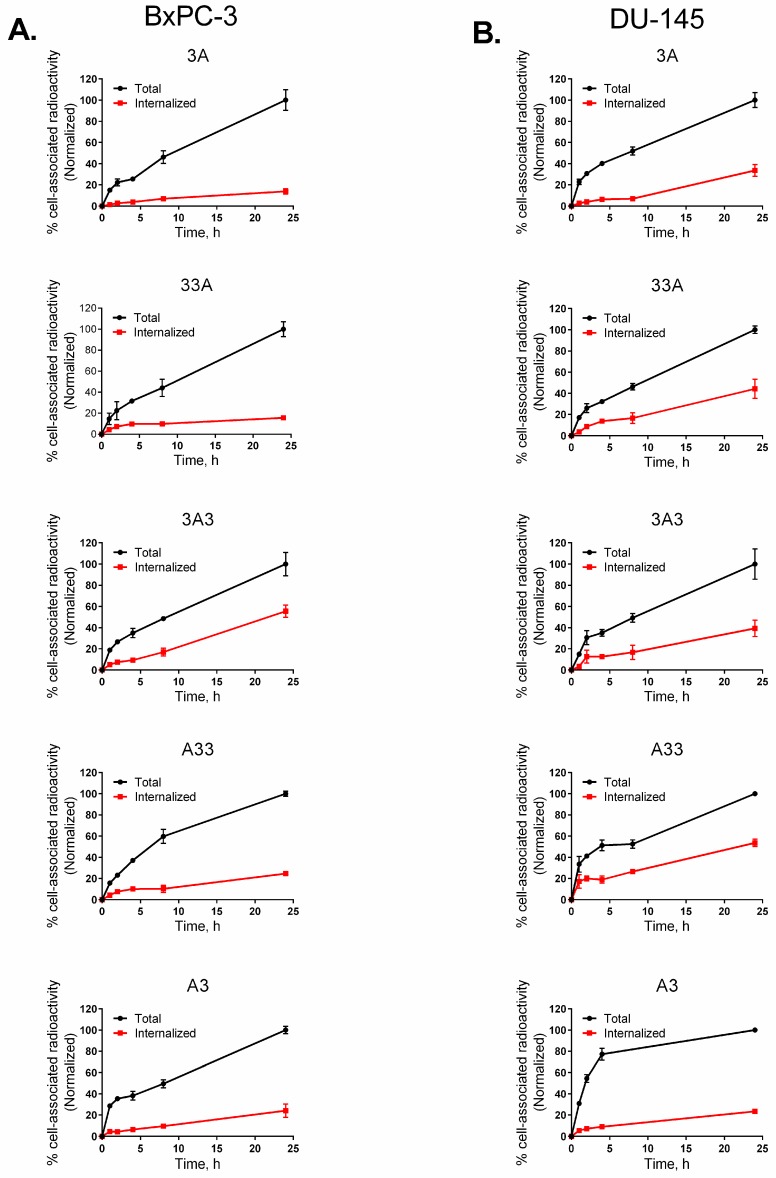
Cellular processing of different ^111^In-labelled ABD-fused anti-HER3 affibody molecules by HER3-expressing BxPC-3 (**A**) and DU-145 (**B**) cell lines with up to 24 h of continuous incubation. Data were normalized to the maximum uptake in each cell line. Data is presented as an average ± SD (*n* = 3).

Results from the assessment of cellular processing and internalization of the radiolabelled anti-HER3 constructs on different cell lines are shown in Figure 5 and Figure S5. In general, there was a similarity in the pattern of processing in all four tested cell lines. All constructs showed a moderate but continuous growth of cell-bound activity up to 24 h of incubation. Among all studied constructs, 3A3 demonstrated the fastest increase of cell-associated activity in all cell lines. The internalization of all conjugates also increased during the whole experiment, although at different rates. Among all studied constructs 3A3 demonstrated the highest rate of internalization in all studied HER3-expressing cell lines. The rate of internalization of 33A was comparable to that of 3A3 in the DU-145, LS174T, and MCF-7 cells but not in the BxPC-3 cells. A common finding from all studied cell lines was the low total cellular uptake of A3. The internalization rate of A3 was also the lowest compared to other studied constructs.

### 3.8. In Vivo Studies

Data concerning tumour targeting and biodistribution of labelled constructs 1, 6, and 24 h p.i. in BxPC-3 xenografted BALB/c-nu/nu mice is represented in Table 3.

Overall, the study demonstrated a strong influence of composition of ABD-fused HER3-targeting agents on overall biodistribution. Most obvious was the influence on activity uptake in blood, tumours, excretory organs (kidney and liver), and organs with endogenous expression of murine ErbB3 (particularly the liver). It is apparent that ABD fusion resulted in prolonged retention in the blood for all constructs, however at different levels. All studied constructs demonstrated comparable blood-associated activity 1 h p.i. except for the 3A construct, which had significantly higher activity. The dimeric constructs, where the ABD scaffold was located on either of the termini (33A and A33), showed faster clearance from the blood between 1 and 24 h p.i. The blood-associated activity was reduced 23-fold for 33A and 10-fold for A33, but only ca. 3–3.5-fold for the other studied constructs. Regardless, 3A still demonstrated significantly higher retention of activity in the blood up to 24 h p.i. compared to the other constructs. A similar effect was also observed for the monomeric construct A3 with the ABD relocated to the N-terminus of the anti-HER3 affibody molecule.

The rapid washout of 33A from the blood was accompanied with high activity accumulation in the tumour and liver. No similar effect was observed for construct A33. Despite its longer retention in the circulation compared to 33A, the monovalent 3A construct had significantly lower accumulation in the tumour 6 h p.i. However, there was no significant difference in the tumour uptake between 3A and 33A by the end of the experiment. Moreover, 3A demonstrated significantly lower activity accumulation in the liver compared to 33A throughout the experiment. On the other hand, the tumour accumulated activity of both A33 and A3 was the lowest (*p* < 0.05) among all studied constructs 24 h p.i. 

Changing the position of ABD from C-terminus in 3A and 33A to N-terminus in A3 and A33 resulted in significantly lower uptake in the kidneys at all studied time points. Throughout the study the previously investigated format 3A3 demonstrated moderate uptake in tumour and kidneys and somewhat lower liver uptake compared to the other constructs.

## 4. Discussion

HER3 overexpression in malignant tumours has been associated with resistance to targeted therapy and poor overall survival. MAbs directed against HER3 (e.g., seribantumab) have been investigated in the clinics in combination with different therapies for treatment of various types of cancers and demonstrated promising results [34]. Affibody molecules represent a non-immunoglobulin-based alternative to mAbs. Plasma half-life extension through genetic fusion of affibody molecules to ABD significantly increases bioavailability of affibody-based targeting agents [23]. It is worth mentioning that the size of the affibody-ABD fused proteins bound to albumin is ca. 80 kDa, approximately only half the size of full-length mAbs (ca. 150 kDa). A small size would mean increased tumour tissue penetration and homogeneity of distribution compared to mAbs [35]. In addition, binding to albumin enables maintaining a sufficiently high concentration of the administered drug in the blood and extracellular space, which could increase potency and improve patient compliance by allowing fewer administrations. We and others have demonstrated the feasibility of HER3 targeting using anti-HER3 affibody molecules [22,36] and bivalent HER3 targeting constructs, with two HER3-specific affibody molecules flanking the ABD demonstrating potent anti-proliferative effects both in vitro and in vivo [27,28]. In the present study, we have designed and explored four novel formats of anti-HER3 affibody molecules fused to ABD (3A, 33A, A3, A33) and the previously investigated 3A3 with the aim to identify an optimal construct design.

All new constructs demonstrated high binding affinity as well as specificity to HER3 receptors. Retaining the capacity to bind HER3 with high affinity is essential as many cancers have low to moderate levels of HER3 expression. Binding of all newly designed affibody constructs was found to inhibit the heregulin-induced phosphorylation in HER3-expressing BxPC-3 and DU-145 cell lines with similar efficiency. Surprisingly, the signal in BxPC-3 negative control was higher in comparison with the DU-145 negative control (Figure 3A). This might be due to the autocrine properties of BxPC-3 cells secreting heregulin as well as other growth factors [37,38]. The ability to self-induce phosphorylation is thought to be important for HER3-associated resistance mechanisms to anti-HER treatments. We have previously observed that absolute inhibition of cell proliferation using a construct similar to 3A3 in vitro was not possible due to this autocrine effect [27]. Nevertheless, inhibition of growth of HER3-expressing cancer cells was efficient and a pilot in vivo therapy study confirmed that it was possible to delay growth of autocrine BxPC-3 xenografts using this construct [27].

Site-specific labelling of affibody molecules with indium-111 via DOTA facilitated comparative evaluation of the different constructs both in vitro and in vivo. Binding of all constructs to HER3 receptors was preserved after radiolabelling as shown in Figure 4.

Cell lines with low to high HER3 expression levels were included in this study. It was demonstrated in clinical studies that not only HER3 expression but also levels of heregulin and HER2 expression influence therapeutic outcome for anti-HER3 treatment [39]. Results from the cellular processing experiments revealed that the format of ABD-fused anti-HER3 affibody molecules had an apparent influence on the processing and internalization of targeting agents after binding to HER3-expressing cells Figure 5 and Figure S5. However, no common pattern of internalization was observed, and different constructs behaved differently in the studied panel of cell lines. This is in line with previous reports on the monomeric anti-HER3 affibody Z_HER3:08699_, which was highly internalized by the cells with high (BT474, (25 ± 2) × 10^3^ receptors/cell, BxPC-3, (12 ± 2) × 10^3^ receptors/cell) HER3 expression but not by the cells with moderate HER3-expression (LS174T, (8.0 ± 0.6) × 10^3^ receptors/cell) [21]. One speculation is that the observed disparity in processing and internalization of HER3-binders in different cell lines might partly be due to differences in levels of HER3 production and expression [27]. Another contributing factor could be the co-expression of the HER family receptors EGFR (epidermal growth factor receptor) and HER2 and the formation of different heterodimers with HER3, which may be involved in altering the internalization patterns of different conjugates. For example, Barta and co-workers observed earlier that binding of affibody molecules targeting HER2 in DU-145 and SKOV-3 cells were influenced by the level of co-expression of EGFR in these cell lines [40]. 

Furthermore, results from the cellular processing have also shown that the HER3-targeting constructs were processed differently by the same cell line. As the level of target expression within an individual cell line is equal, differences in processing of the studied constructs can probably be attributed to a varying ability to trigger HER3 internalization. The bivalent 3A3 may for example induce HER3 internalization in a way similar to that observed for HER3 targeting mAbs, thus resulting in higher internalization rate of this format compared to other constructs [41]. Although it is beyond the scope of this study, increased internalization of some formats may render these constructs promising candidates for payload delivery of cytotoxic agents and small drug molecules to tumour cells.

Due to the extremely high concentration of albumin in blood (around 40 mg/mL), only a moderate affinity for serum is needed for extending the in vivo half-life [42]. All tested conjugates demonstrated high affinity to albumin (from subnanomolar to low picomolar). As expected, the circulatory half-life was prolonged for all conjugates by the inclusion of ABD (Table 3). Activity concentration in blood 1 h p.i. was 20–30-fold higher for the studied constructs than for anti-HER3 monomer [22]. The magnitude of this enhancement was in accordance with the effect observed earlier for both monomeric and dimeric anti-HER2 and anti-HER3 affibody molecules, fused either on the N- or C-termini of ABD [27,43,44]. Surprisingly, the effect on circulatory half-life of the bivalent formats with terminal ABD, 33A and A33 was not as prominent compared to the other studied constructs. The elimination of 33A and A33 from blood was faster compared to that of 3A, 3A3, and A3. This unexpected behaviour might be associated with compromised binding to albumin as a result of structure-related steric hindrance caused by two consecutively coupled HER3 targeting monomers. Decreased binding to albumin might render freely circulating 33A and A33 more efficiently sequestered from the blood by murine ErbB3-expressing tissues. The significantly higher uptake of labelled 33A and A33 in the liver and spleen supports this hypothesis.

The comparative biodistribution study showed the capacity of ABD-fused proteins to accumulate in HER3-expressing tumours. Interestingly, the fusion protein 33A provided higher tumour uptake values compared to other conjugates at as early as 6 h p.i., likely due to elevated fraction of freely circulating 33A (i.e., not bound to albumin) that also leads to more rapid blood clearance. On the other hand, 3A and 3A3 conjugates had a longer residence time in blood than 33A and had better bioavailability 24 h p.i. However, this was not the case for A33 where rapid washout from the circulation was not accompanied by increased accumulation in the tumour. In fact, more A33 was directed towards the kidneys and excreted. The A3 format also demonstrated elevated renal excretion and low tumour accumulation. The observed low accumulation of A3 in the tumour is not in good agreement with the measured high binding affinity to recombinant HER3 (subnanomolar) in the biosensor assay (Table 1). However, A3 also had the lowest binding to HER3-expressing cells in vitro (Figure 4). The discrepancy between the affinity using immobilized recombinant receptors and both in vitro and in vivo assays suggests that the ligand–receptor interaction is more complicated in the latter case, and that sorting of targeting agents might not be addressed adequately using a single criterion. Together, results from the comparative biodistribution study indicate that placing ABD at the N-terminus of HER3-targeting affibody molecules is associated with suboptimal in vivo targeting properties. Moreover, the enhanced plasma half-life of our newly designed 3A3 resulted in reduced renal uptake as well as improved targeting of HER3-expressing BxPC-3 xenografts compared to the previously reported 3A3 variant by Bass et al. [27].

It is especially interesting to compare the 3A3 variant used in this study with the one previously reported by Bass et al. [27]. Despite the fact that such a comparison is not the main aim of this study and that the two variants were not compared head-to-head, we still believe that comparing these two is useful for showing the importance of the structure–properties relationship in drug design. The newly designed 3A3 demonstrated 10-fold higher activity concentration in blood and ca. 1.5-fold lower activity in liver and kidneys 24 h p.i. compared to the previously reported 3A3 by Bass et al. ((6.7 ± 0.8) vs. (0.6 ± 0.2) %ID/g in blood, (8 ± 1) vs. (15 ± 1) %ID/g in liver, and (19 ± 1) vs. (30 ± 4) %ID/g in kidneys). Such significant differences in blood retention between the two constructs cannot solely be explained by batch-to-batch variability of animals. As mentioned above, the newly designed 3A3 used in the current study comprises a shorter G_3_S linker between binding moieties instead of the previously used (S_4_G)_4_ linker. It is likely that potential constraints on the albumin-binding activity of 3A3 were alleviated with the shorter linker, thus enabling stronger binding to serum albumin. Another aspect is the observed difference in tumour uptake between the 3A3 variant used in this study and the previously reported variant ((9 ± 1) vs. (5.3 ± 0.7) %ID/g, 24 h p.i., respectively). One may speculate that such difference may be partially explained by differences in blood retention and overall bioavailability between the two variants. We also cannot exclude that tumour batch-to-batch variability may have contributed to such observed differences, and in future a head-to-head comparison of the two variants is thus warranted in order to draw solid conclusions.

In conclusion, the present study has increased the understanding of the connection between molecular structures and properties for development of smaller HER3-targeting affibody molecules with prolonged half-life. The formats with ABD located on the C-terminus of affibody binding domains (3A and 33A) as well as the redesigned 3A3 format provided the best tumour-targeting properties in vivo. Further evaluation of these promising targeting agents is therefore justified. 

## Figures and Tables

**Table 1:** Biophysical characteristics of different ABD-fused anti-HER3 affibody constructs. HSA: human serum albumin; T_m_: melting temperature; K_D_: equilibrium dissociation constant.

Construct	MW (Da)	T_m_ (°C)	K_D_, HER3 (nM, Mean ± SD)	K_D_, HSA (nM, Mean ± SD)
3A	12,670.9	61.3	0.3 ± 0.03	0.06 ± 0.02
33A	19,601.6	66.1	0.6 ± 0.03	0.24 ± 0.22
3A3	19,601.6	62.4	1.1 ± 0.1	0.68 ± 0.66
A33	19,732.8	62.7	0.8 ± 0.15	0.35 ± 0.24
A3	12,802.1	61.8	0.6 ± 0.15	0.15 ± 0.08

**Table 2:** Radiolabelling yield, radiochemical purity after purification using a NAP-5 size exclusion column, and stability on ethylenediaminetetraacetic acid (EDTA) challenge of different ^111^In-labelled ABD-fused anti-HER3 affibody molecules.

Construct	Radiolabelling Yield (%)	Radiochemical Purity of Conjugates (%)	Protein Associated Activity after 1 h of Incubation with a 500-Fold Molar Excess of EDTA (%)
3A	89 ± 8	99 ± 1	99.6 ± 0.3
33A	85.1 ± 0.1	98.6 ± 0.8	99.4 ± 0.1
3A3	51 ± 12	98 ± 1	98 ± 1
A33	28 ± 6	97 ± 2	96 ± 1
A3	45 ± 4	97 ± 2	96 ± 1

**Table 3:** Biodistribution of ^111^In–labelled ABD-fused anti-HER3 affibody molecules in female BALB/c-nu/nu mice with BxPC-3 xenografts 1, 6, and 24 h after intravenous injection. The measured radioactivity of different organs is expressed as % of injected dose per gram tissue (%ID/g), and presented as an average value from four animals ± SD. GI tract: gastrointestinal tract.

Organ	3A	33A	3A3	A33	A3
**1 h**					
**Blood**	33 ± 3 *^c,d^*	25 ± 2	24 ± 1	20.2 ± 0.9	21 ± 1
**Salivary gland**	3.6 ± 1.0	2.8 ± 0.4	2.6 ± 0.6	2.4 ± 0.5	2.7 ± 0.5
**Lung**	12 ± 2	10 ± 2 *^f^*	8 ± 1	8 ± 2	8.2 ± 0.8
**Liver**	7 ± 2	13 ± 1 *^g^*	7 ± 1	9 ± 2	5.8 ± 0.6
**Spleen**	6 ± 1	8 ± 2	5.0 ± 0.6	5.9 ± 0.4 ^j^	4.0 ± 0.2
**Stomach**	2.0 ± 0.3	1.9 ± 0.5	1.5 ± 0.3	1.5 ± 0.4	1.6 ± 0.2
**Small intestine**	5 ± 1	5.5 ± 0.4	3.6 ± 0.3	3.4 ± 0.6	3.6 ± 0.8
**Kidney**	10 ± 2 *^b,c,d^*	11 ± 1 *^e,f,g^*	22 ± 2 *^h^*	41 ± 3	33 ± 2
**Tumour**	4.1 ± 0.5	4.3 ± 0.4	3.2 ± 0.7	4 ± 1	4 ± 2
**Muscle**	1.0 ± 0.1	0.9 ± 0.2	0.9 ± 0.1	0.7 ± 0.1	0.7 ± 0.1
**Bone**	2.4 ± 0.3	2.4 ± 0.4	1.9 ± 0.7	1.7 ± 0.5	2.0 ± 0.9
GI tract *	4 ± 1	4.7 ± 0.7 *^k^*	3.1 ± 0.7	2.7 ± 0.3	2.9 ± 0.5
Carcass *	32 ± 6	30 ± 2 *^f,k^*	27 ± 2	21 ± 2	25.2 ± 0.7
**6 h**					
**Blood**	19 ± 2 *^a,c,k^*	9.5 ± 0.8 *^k^*	14 ± 2 *^k^*	9 ± 1 *^k^*	16 ± 2
**Salivary gland**	4.3 ± 0.4 *^c^*	3.35 ± 0.08 *^f^*	3.2 ± 0.5 *^h^*	2.1 ± 0.2	3.8 ± 0.7
**Lung**	9.0 ± 0.6 *^a,c^*	6.8 ± 1.0 *^f^*	6 ± 1	4.3 ± 0.6 *^j^*	8.4 ± 0.9
**Liver**	6.2 ± 0.3 *^a^*	19 ± 1.5 *^e,f,g,k^*	6.6 ± 0.6	9.1 ± 0.6	5.6 ± 0.3
**Spleen**	4.4 ± 0.8 *^a^*	14.0 ± 0.3 *^e,f,g,k^*	5.7 ± 0.7	8 ± 2	5 ± 1
**Stomach**	2.5 ± 0.4 *^k^*	2.5 ± 0.2 *^f^*	1.6 ± 0.3	1.5 ± 0.2 *^j^*	2.24 ± 0.09
**Small intestine**	4.3 ± 0.7	9 ± 3	3.5 ± 0.9	4 ± 1	4.2 ± 0.7
**Kidney**	9 ± 1 *^a,b,c,d^*	12.0 ± 0.6 *^f,g,k^*	21 ± 2 *^h^*	41 ± 4	44 ± 8
**Tumour**	8 ± 2 *^a^*	13 ± 2 *^e,f^*	7 ± 1	7 ± 1	8 ± 1
**Muscle**	1.6 ± 0.2 *^c,k^*	1.08 ± 0.09	1.0 ± 0.2	0.72 ± 0.07 *^k^*	1.3 ± 0.1
**Bone**	2.0 ± 0.2	4 ± 1	1.8 ± 0.4	1.68 ± 0.07	2.4 ± 0.5
GI tract *	5.8 ± 0.7 *^a^*	8.7 ± 0.8 *^f,g^*	4 ± 1	3.97 ± 0.07 *^k^*	4.1 ± 0.2
Carcass *	40 ± 3 *^c^*	36 ± 3 *^f^*	32 ± 2 *^h^*	20.8 ± 0.1 *^j,k^*	33.9 ± 0.3
**24 h**					
**Blood**	10 ± 2 *^a,c,l,m^*	1.0 ± 0.2 *^e,g,l,m^*	6.7 ± 0.8 *^h,l,m^*	2.0 ± 0.3 *^j,l,m^*	6.3 ± 0.6 *^l,m^*
**Salivary gland**	4.4 ± 0.7	2.5 ± 0.5	3.3 ± 0.2 ^h^	2.1 ± 0.3 ^j^	3.2 ± 0.2
**Lung**	5.8 ± 0.7 *^c,l,m^*	2.7 ± 0.8 *^l,m^*	4.5 ± 0.8	1.9 ± 0.3 *^l,m^*	3.6 ± 0.7 *^l,m^*
**Liver**	5.9 ± 0.6 *^a,c^*	19 ± 2 *^e,f,g^*	8 ± 1	10.1 ± 0.3 *^j^*	5.3 ± 0.6
**Spleen**	5.0 ± 0.8	15 ± 5	6.6 ± 0.8	6.7 ± 0.8	3.4 ± 0.7
**Stomach**	2.3 ± 0.3 *^c,m^*	1.7 ± 0.7	1.7 ± 0.2	1.2 ± 0.1	1.7 ± 0.3
**Small intestine**	6 ± 1	7 ± 3	4.4 ± 0.3	3.3 ± 0.2	4.1 ± 0.6
**Kidney**	7.5 ± 0.7 *^b,c,d^*	9 ± 2 *^f,g^*	19 ± 1 *^h,i^*	32.7 ± 0.6 *^l,m^*	31 ± 3
**Tumour**	9 ± 2 *^m^*	12 ± 2 *^f,m^*	9 ± 1 *^h,i^*	6.5 ± 0.9	5.9 ± 0.4
**Muscle**	1.2 ± 0.2 *^l^*	0.6 ± 0.1	1.0 ± 0.1	0.6 ± 0.1	0.9 ± 0.2
**Bone**	2.3 ± 0.4	3 ± 1	1.7 ± 0.6	1.7 ± 0.3 *^l^*	1.5 ± 0.3
GI tract *	5.8 ± 0.8 *^c,m^*	6 ± 1	4.1 ± 0.4	3.2 ± 0.3 *^m^*	5.2 ± 0.4
Carcass *	36 ± 3 *^c^*	27 ± 3 *^l^*	29 ± 3	16 ± 1 *^l^*	25 ± 2

* Data for gastro-intestinal tract with content and carcass are presented as percentage of injected radioactivity per whole sample. Data were assessed by one-way ANOVA with Bonferronni correction for multiple comparisons in order to determine significant differences between groups (*p* < 0.05). *^a^*—3A vs. 33A at this time point; *^b^*—3A vs. 3A3 at this time point; *^c^*—3A vs. A33 at this time point; *^d^*—3A vs. A3 at this time point; *^e^*—33A vs. 3A3 at this time point; *^f^*—33A vs. A33 at this time point; *^g^*—33A vs. A3 at this time point; *^h^*—3A3 vs. A33 at this time point; *^i^*—3A3 vs. A3 at this time point; *^j^*—A33 vs. A3 at this time point; *^k^*—1 h p.i. vs. 6 h p.i. for this conjugate; *^l^*—6 vs. 24 h p.i. for this conjugate; *^m^*—1 vs. 24 h p.i. for this conjugate.

## Supplementary Materials

The following are available online at http://www.mdpi.com/2073-4409/7/10/164/s1, Figure S1: Purity determination. Figure S2: Mass determination. Figure S3: Analysis of thermal stability and refolding capacity of DOTA-conjugated constructs. Figure S4: Analysis of binding affinity to HSA. Figure S5: Cellular processing of different ^111^In-labelled ABD-fused anti-HER3 affibody molecules by LS174T and MCF-7 cells.
