# Supplementary material for: Influence of Molecular Design on the Targeting Properties of ABD-Fused Mono- and Bi-Valent Anti-HER3 Affibody Therapeutic Constructs
Source: Cells. 2018 Oct 11;7(10):164. doi: 10.3390/cells7100164 (PMC6210767; doi:10.3390/cells7100164)
Supplement: Supplementary file 1 [file cells-07-00164-s001.pdf]

Supplementary Material

Influence of Molecular Design on the Targeting Properties of ABD-Fused Mono- and Bi-Valent Anti-HER3 Affibody Therapeutic Constructs

Mohamed Altai [1,2,*,†], Charles Dahlsson Leitao [3,†], Sara S. Rinne [2], Anzhelika Vorobyeva [1], Christina Atterby [1], Stefan Ståhl [3], Vladimir Tolmachev [1], John Löfblom [3], Anna Orlova [2,4]

1 Department of Immunology, Genetics and Pathology, Uppsala University, 751 85 Uppsala, Sweden; anzhelika.vorobyeva@igp.uu.se (A.V.); christina.atterby@igp.uu.se (C.A.); Vladimir.tolmachev@igp.uu.se (V.T.)

2 Department of Medicinal Chemistry, Uppsala University, 751 23 Uppsala, Sweden; sara.rinne@ilk.uu.se (S.S.R.); anna.orlova@ilk.uu.se (A.O.)

3 Department of Protein Science, School of Engineering Sciences in Chemistry, Biotechnology and Health, KTH Royal Institute of Technology, 106 91 Stockholm, Sweden; chdl@kth.se (C.D.L.); stefans@biotech.kth.se (S.S.); lofblom@kth.se (J.L.)

4 Science for Life Laboratory, Uppsala University, 752 37 Uppsala, Sweden

* Correspondence: Mohamed.altai@igp.uu.se; Tel.: +46-18-471-3414; Fax: + 46-18-471-3432

† These authors contributed equally to this work.

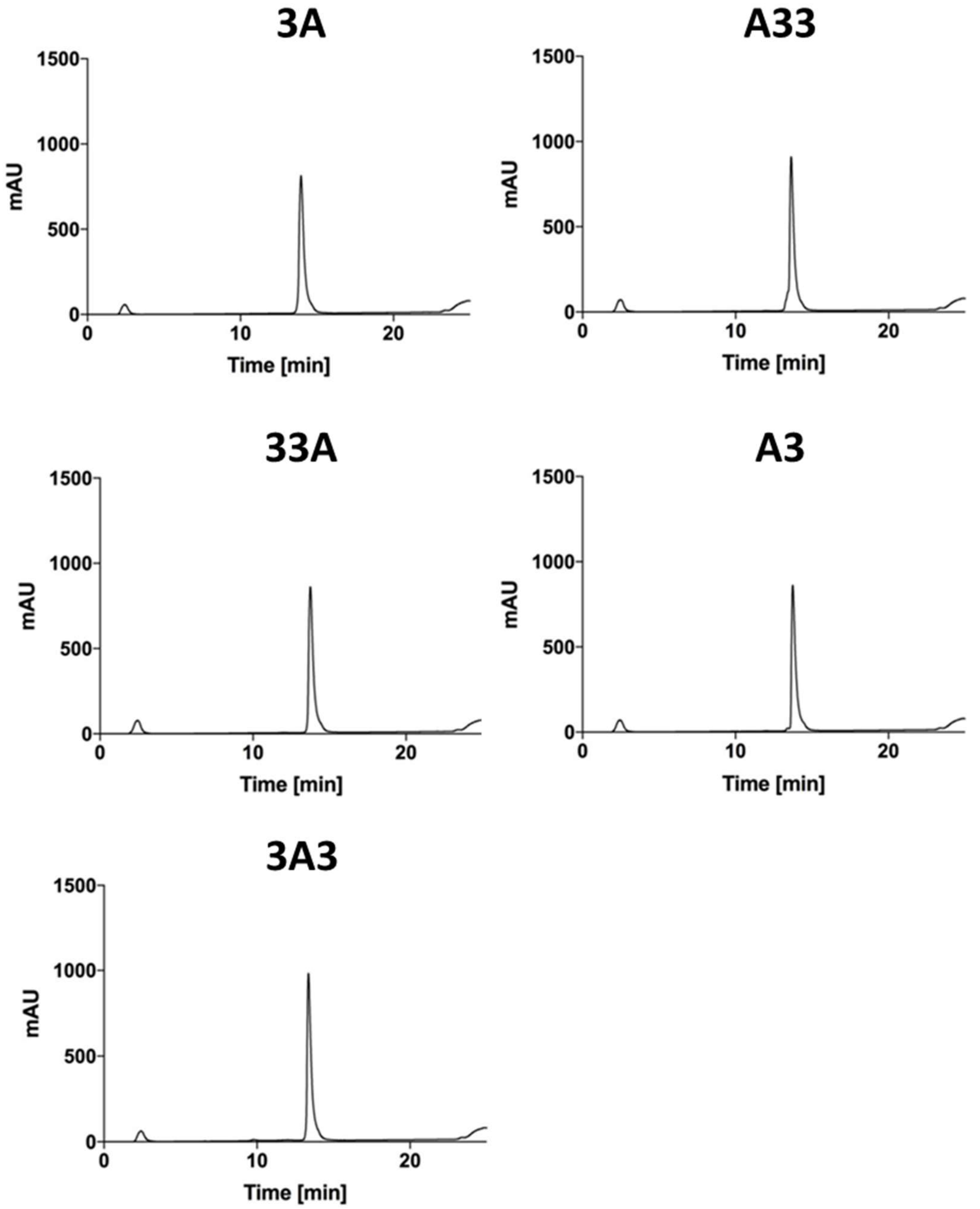

Figure S1: Purity determination. The purity of the five constructs was evaluated by absorbance measurement at 220 nm using RP-HPLC.

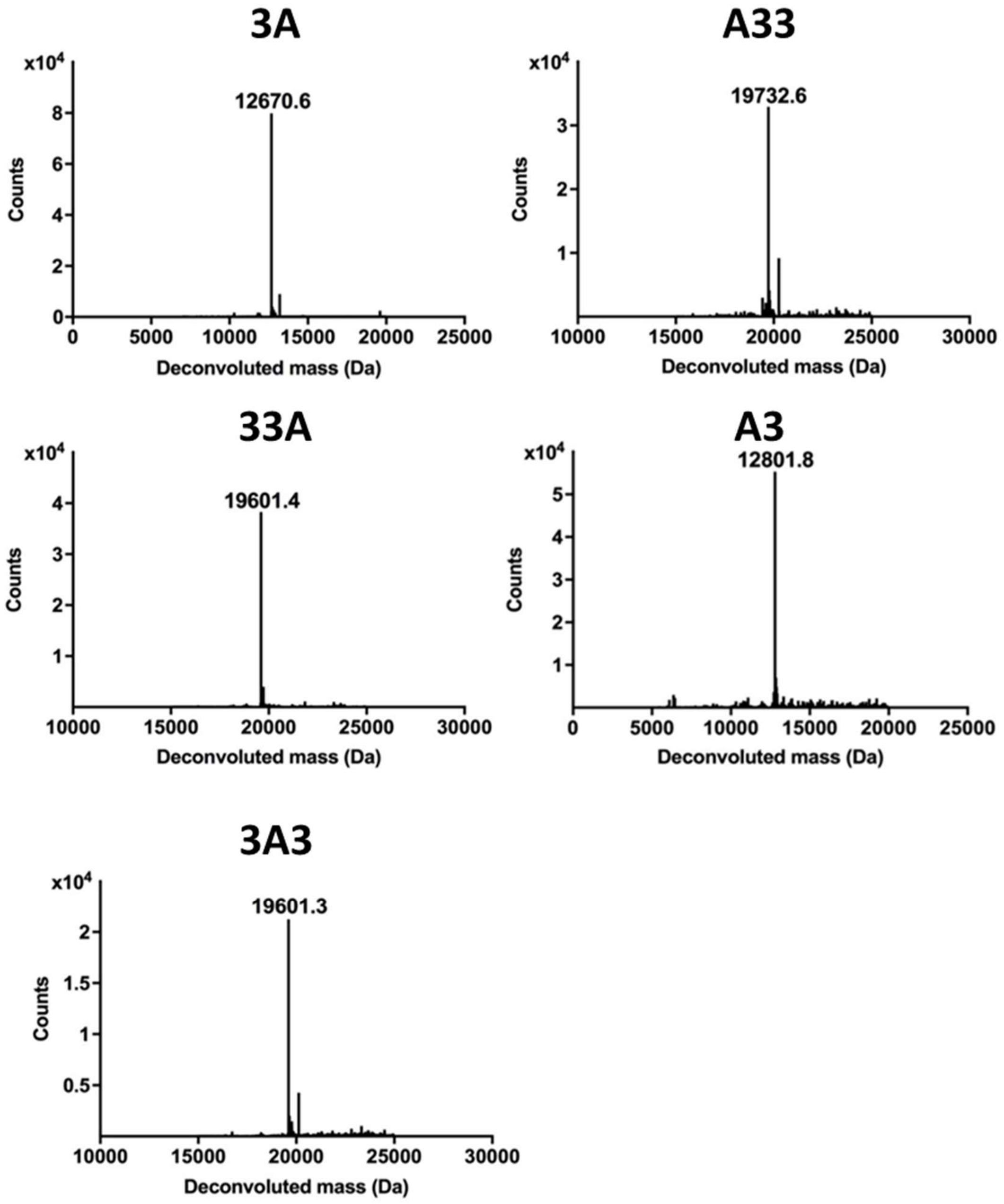

Figure S2: Mass determination. Results from ESI-MS confirming the identity of the constructs. The experimental molecular masses of the observed peaks are in accordance with the theoretical masses shown in Table 1.

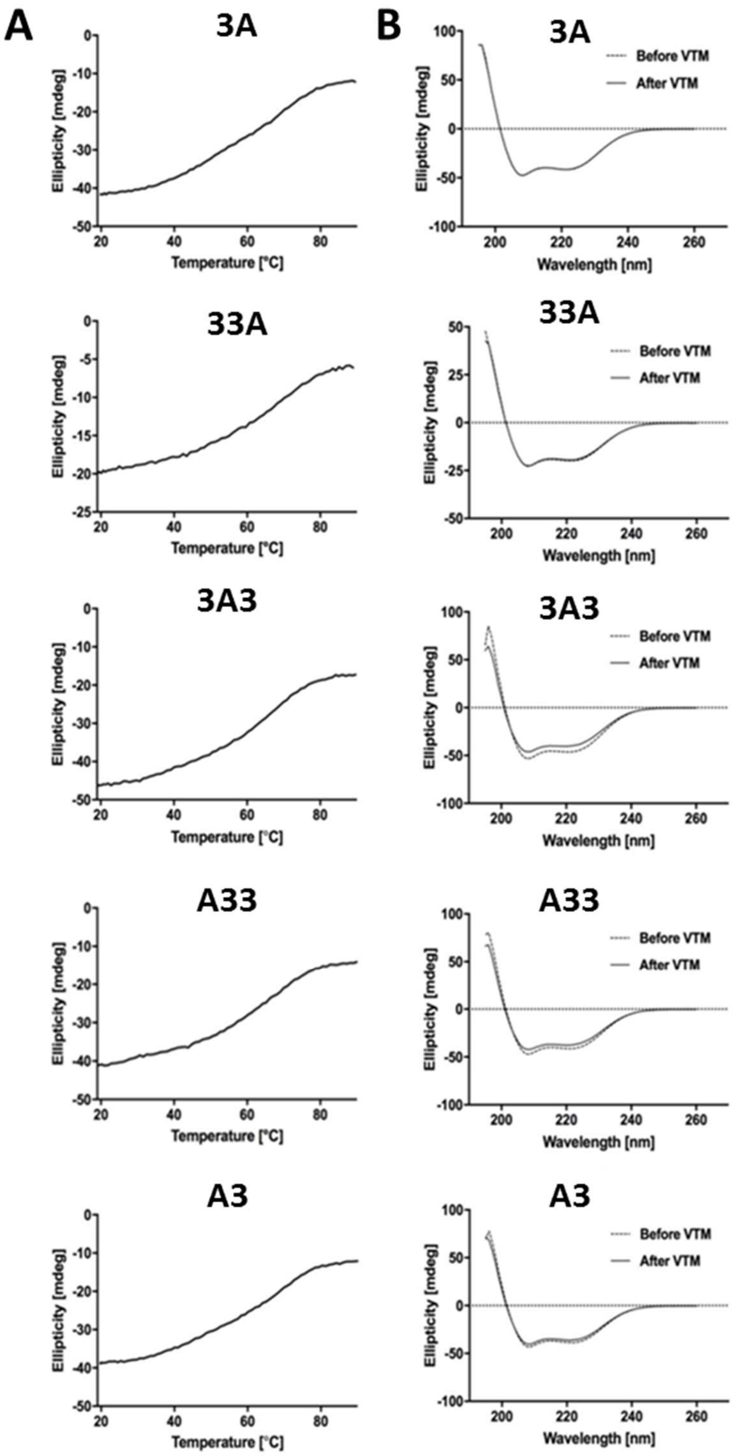

Figure S3: Analysis of thermal stability and refolding capacity of DOTA-conjugated constructs. A) overlay of circular dichroism spectra (195-260 nm) before and after thermal denaturation. B) Variable temperature measurement (VTM) spectra obtained at 221 nm while heating the sample from 20 °C to 90 °C. Melting temperatures (T_m) were determined by fitting the curves using a Boltzmann Sigmoidal model. The determined T_m values for the five constructs are presented in Table 1.

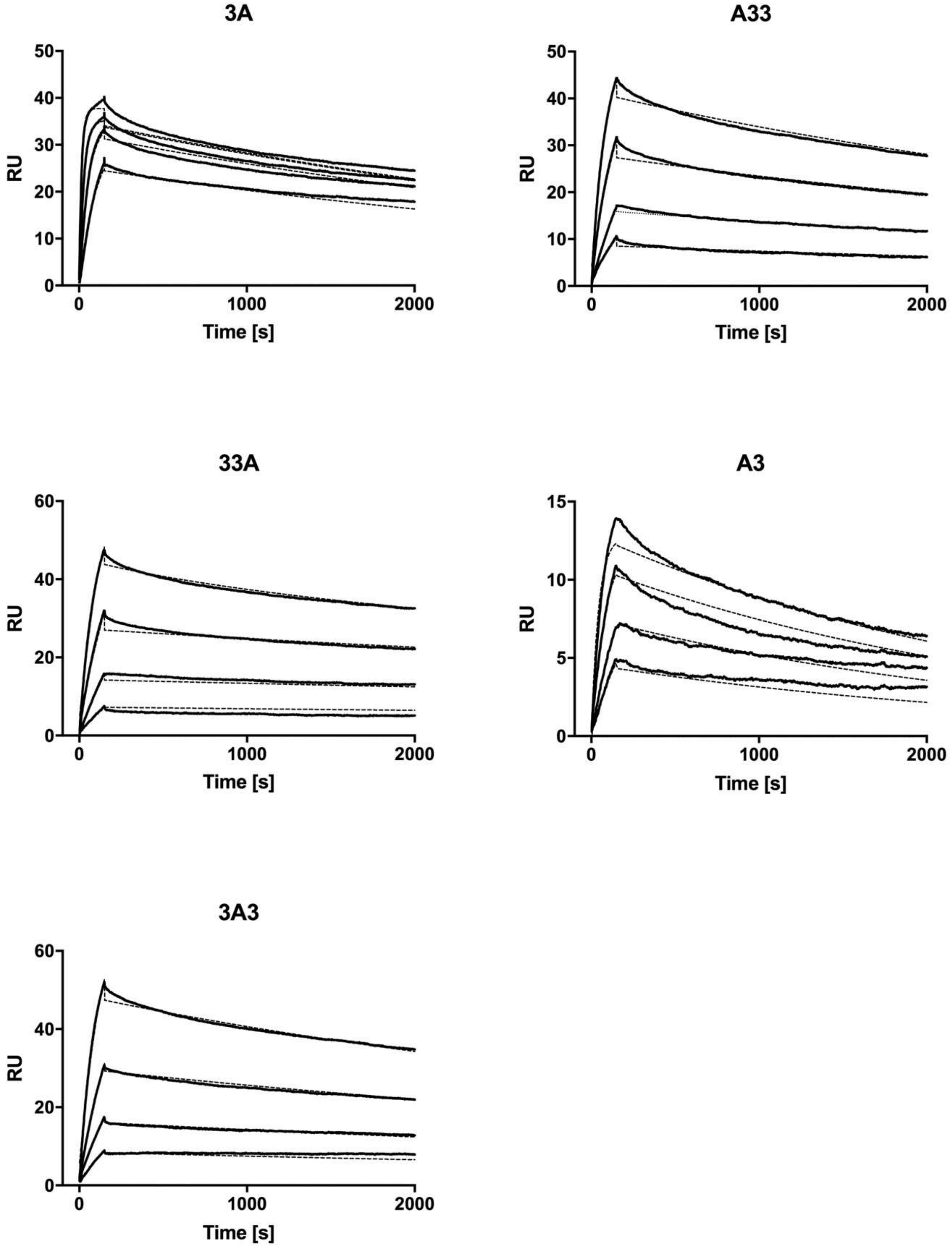

Figure S4: Analysis of binding affinity to HSA. Representative experimental sensorgrams (solid) with fitted curves (dashed) from SPR analysis for the five DOTA-conjugated ABD-fused anti-HER3 affibody constructs. Immobilized HSA was subjected to four concentrations of each construct (1.5625, 3.125, 6.25 and 12.5 nM). Monovalent affinities to HSA, based on a Langmuir 1:1 model, are presented in Table 1.

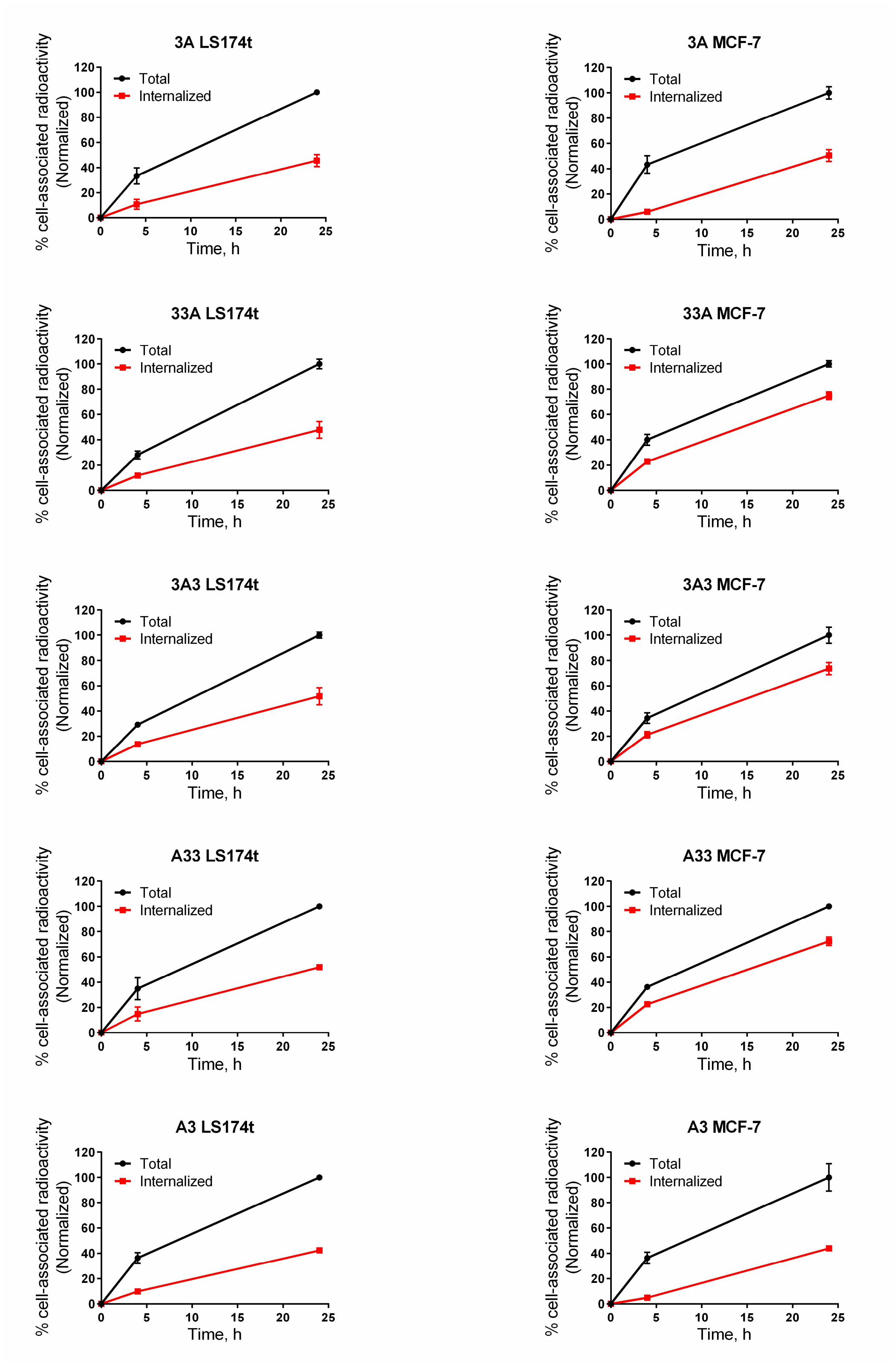

Figure S5: Cellular processing of different ^{111}In-labeled ABD-fused anti-HER3 affibody molecules by LS174T (left) and MCF-7 (right). HER3-expressing cell lines up to 24 h after continuous incubation. Data were normalized to the maximum uptake.
